# Endoscopic removal of a challenging gastric foreign body causing unexplained abdominal pain

**DOI:** 10.1055/a-2466-9854

**Published:** 2024-12-04

**Authors:** Huijie Wu, Xiaolu Lin, Wanyin Deng

**Affiliations:** 1 117861 Digestive Endoscopy Center, Fujian Provincial Hospital, Fuzhou University Affiliated Provincial Hospital, Fuzhou, China; 2 Shengli Clinical Medical College of Fujian Medical University, Fuzhou, China

A 33-year-old woman presented with a 3-month history of upper abdominal pain, which was exacerbated by bending or twisting. Blood tests revealed no abnormalities, and pharmacological interventions failed to alleviate symptoms. Gastroscopy at a local hospital had shown a 0.6-cm firm protrusion on the greater curvature of the gastric antrum, without discoloration or texture changes, that was suspicious for a submucosal tumor (SMT) ( Fig. 1 **a** ). However, gastroscopy at our center revealed that the lesion had shrunk in size compared to the initial assessment, casting doubt on the SMT diagnosis ( Fig. 1 **b** ). Subsequent endoscopic ultrasound (EUS) showed a cordlike hyperechoic shadow penetrating the gastric muscular layer, suggesting the presence of a foreign body ( Fig. 1 **c** ). Despite the patient denying ingestion of any foreign object, a computed tomography scan corroborated the EUS findings ( Fig. 1 **d** ). Given the patient’s persistent pain and the shrinkage of the lesion, endoscopic submucosal dissection (ESD) was carried out with her consent ( Video 1 ). After the lesion had been marked and a circular incision had been made, intraoperative EUS helped to locate the deeply embedded foreign body ( Fig. 2 **a–c** ). The foreign body was carefully extracted and found to be a sharp, 2-cm metallic object (iron wire), which had caused the gastric mucosal lesion to resemble an SMT ( Fig. 2 **d,e** ). The wound was closed with endoscopic clips, and no bleeding was observed ( Fig. 2 **f** ). The patient was discharged 5 days after the operation, with no recurrence of pain reported at the 2-month follow-up.

**Fig. 1:**
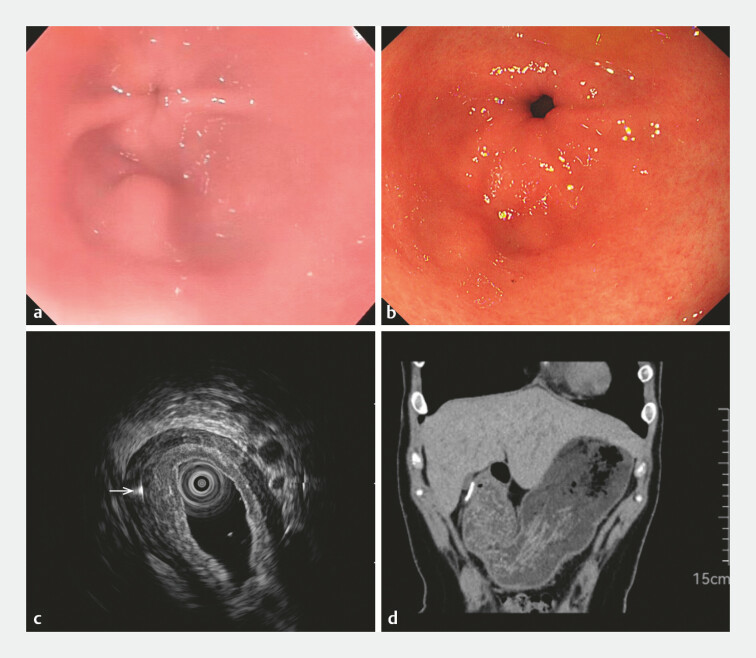
**a** Gastroscopy at a local hospital had shown a 0.6-cm protrusion on the greater curvature of the gastric antrum, that was suspected to be a submucosal tumor. **b** Gastroscopy at our center revealed a reduction in lesion size. **c** Endoscopic ultrasound showed a cordlike hyperechoic shadow penetrating the gastric muscular layer (arrow), suggestive of a foreign body. **d** Computed tomography scan confirmed the presence of a foreign body.

**Fig. 2:**
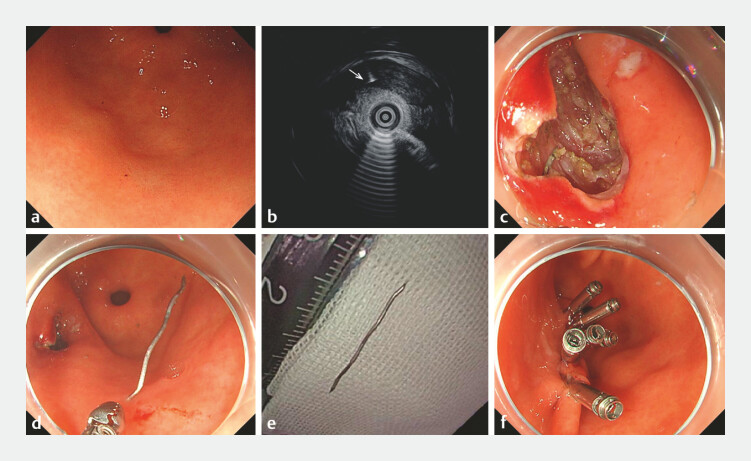
**a** Gastroscopy showed a reduction in lesion size compared to the initial assessment. **b** A cordlike hyperechoic shadow (arrow) in the muscular layer of the gastric antrum was accurately identified and marked under endoscopic ultrasound guidance. **c** The foreign body was deeply embedded in the muscular layer. **d** The foreign body was carefully clamped with foreign body forceps. **e** Successfully extracted foreign body that proved to be a 2-cm length of iron wire. **f** The wound after closure with endoscopic clips.

Successful removal of a foreign body from the gastric antrum using endoscopic submucosal dissection under endoscopic ultrasound guidance.Video 1

Ingestion of a foreign body is common and most cases are asymptomatic; only a few foreign bodies cause symptoms and complications 1 2 3 . In this rare case, EUS was crucial in detecting the foreign body and guiding its successful removal via ESD, highlighting the diagnostic challenges of atypical foreign bodies and the value of applying multiple endoscopic techniques for precise, minimally invasive treatment.

Endoscopy_UCTN_Code_TTT_1AO_2AL

## Notes

Endoscopy E-Videos https://eref.thieme.de/e-videos
*E-Videos* is an open access online section of the journal *Endoscopy* , reporting on interesting cases and new techniques in gastroenterological endoscopy. All papers include a high-quality video and are published with a Creative Commons CC-BY license. Endoscopy E-Videos qualify for HINARI discounts and waivers and eligibility is automatically checked during the submission process. We grant 100% waivers to articles whose corresponding authors are based in Group A countries and 50% waivers to those who are based in Group B countries as classified by Research4Life (see: https://www.research4life.org/access/eligibility/ ). This section has its own submission website at https://mc.manuscriptcentral.com/e-videos .
